# Measuring community engagement and trust in community health workers in Haiti, Malawi, and Rwanda: a cross-sectional study

**DOI:** 10.7189/jogh.16.04217

**Published:** 2026-07-24

**Authors:** Stephanie Armbruster, Stefanie A Joseph, Kobel Dubique, Erick Baganizi, Frank Gondwe, Nadege Belizaire, Maurice Junior Chery, Peterson Abnis Faure, Tumusime Musafiri, Benson Chabwera, Jean Claude Mugunga, Mary Clisbee, Dale A Barnhart, Bethany Hedt-Gauthier, Fabien Munyaneza

**Affiliations:** 1 Department of Biostatistics, Harvard T.H. Chan School of Public Health, Boston, Massachusetts, USA; 2 Partners In Health, Boston, Massachusetts, USA; 3 Partners In Health/Zanmi Lasante, Mirebalais, Haiti; 4 Partners In Health/Inshuti Mu Buzima, Kigali, Rwanda; 5 Partners In Health/Abwenzi Pa za Umoyo, Neno, Malawi; 6 Independent Researcher, Malawi; 7 Department of Maternal and Child Health and Department of Biostatistics, Gillings School of Global Public Health, University of North Carolina at Chapel Hill, Chapel Hill, North Carolina, USA; 8 Swiss Tropical and Public Health Institute, Allschwil, Switzerland; 9 University of Basel, Basel, Switzerland

**Keywords:** community participation, community health workers, trust, professional-patient relationship, health equity, Haiti, Malawi, Rwanda

## Abstract

**Background:**

Community health workers (CHWs) increase the accessibility of institutional health systems by building trust in communities. We assessed community members’ trust in CHWs in Haiti, Malawi, and Rwanda. We explored whether potentially modifiable factors, including sex concordance between community members and CHWs and time spent by CHWs engaging with their community, are associated with increased trust in CHWs.

**Methods:**

Between June and October 2023, we used two-stage sampling to conduct a cross-sectional survey of 1920 community members, cared for by Partners In Health. We multiplied the frequency and duration of self-reported interactions with CHWs to calculate cumulative self-engagement and cumulative perceived engagement between a CHW and others in their community over the past six months. Community members who interacted with their CHW at least once in the past six months answered the Trust-in-CHW Scale. We compared engagement levels by country using a Rao-Scott χ^2^ test and explored the association between sex and sex concordance, CHW engagement, and trust, by country using clustering-adjusted regression models.

**Results:**

Many community members in Haiti (51.1%), Rwanda (47.2%), and Malawi (5.8%) reported no interaction with their CHW in the past six months (*P* < 0.001). Among those who had interacted at least once, the cumulative self-engagement duration (in hours) was highest in Malawi (mean (x̄) = 3.3 hours), followed by Rwanda (x̄ = 1.2) and Haiti (x̄ = 1.1). Trust in CHWs was also the highest in Malawi (x̄ = 3.6), followed by Rwanda (x̄ = 3.5) and Haiti (x̄ = 3.4). While neither sex nor sex concordance were significantly associated with self-engagement duration or trust, longer cumulative durations of self- and community-engagement were significantly associated with increased trust for all countries.

**Conclusions:**

Increased duration of engagement with CHWs was associated with increased trust. However, many community members reported no meaningful engagement with their CHWs in the past six months. This finding underscores the importance of empowering CHWs to engage with community members.

Many low- and middle-income countries (LMICs) face a shortage of healthcare professionals [1–5]. In these settings, community health workers (CHWs) play a crucial role in bridging the accessibility gap between the institutional healthcare system and communities [2,5]. They provide various health-related services, engage in health education outreach, and contribute to disease prevention efforts [6–9]. Compared to other health professionals, CHWs offer two main advantages. First, because they often serve as volunteers or offer services at low costs, they provide a cost-effective alternative to other healthcare professionals [7]. Second, because they operate within the communities where they reside, CHWs are uniquely positioned to become health role models, foster health promotion, enhance health literacy, and advocate for the needs of their community [5,7,8].

The effectiveness and acceptance of a CHW programme strongly depend on the establishment of frequent interaction and trust between CHWs and community members [3,4,10–14]. The CHWs take on essential instructive and supportive relationships with their communities, including empowering them to take agency in their personal healthcare, adhere to long-term treatments of chronic diseases [8], and care for their newborns [10]. Trust is both a prerequisite for and a product of consistent meaningful engagement [10–12,15]. Trust has been repeatedly identified as a key feature of a strong relationship between a CHW and their community [12,13,15,16]. Trust also fosters more support and understanding for vulnerable populations and strengthens the feeling of social connectedness within communities [11]. Building trust between CHWs and community members may be particularly critical in LMICs, where trust in formal health systems may be diminished [15,17]. When there are mutual trust, competence, transparent communication, honesty, and vulnerability, the relationship between CHWs and the community becomes a cornerstone of effective health service delivery [16,18]. However, CHW engagement and trust are often subgroup-specific, such as for mothers and children, reflecting differences in medical needs and underlying social structures [13].

We conceptualised trust and engagement between CHWs and community members as a bidirectional and mutually reinforcing process (Appendix S1 in the **Online Supplementary Document**). Trust may facilitate future engagement, while repeated and meaningful engagement provides opportunities for trust to develop. However, trust is latent and not directly actionable. Community engagement is an observable lever through which policy can operate. Accordingly, in this study, we adopted a directional analytic focus in which engagement is the modifiable exposure and trust is the observed outcome. This approach does not assume that engagement is the sole determinant of trust, but reflects the practical reality that programmes can adjust engagement opportunities, whereas trust emerges as a relational consequence of multiple interacting factors. Within this framework, we conceptualised the sex of community members and of CHWs and sex concordance between CHWs and community members as contextual confounders that may shape quality and ease of engagement. Shared sex may influence comfort, communication, and social alignment during interactions, thereby potentially modifying the relationship between engagement and trust rather than acting as independent determinants of trust [15].

During the COVID-19 pandemic, governments relied on CHWs to relay critical information and enforce public health measures to manage the outbreak while restricting human-to-human interaction [19]. However, in an environment of stigma, stereotypes, and misconceptions, building and maintaining sustainable trust between CHWs and their communities proved challenging [4,14,20]. The post-COVID-19 context is particularly salient for this framework, as the pandemic disrupted routine CHW-community interactions while simultaneously heightening the importance of trusted intermediaries for health information and service linkage. Examining engagement and trust in this period provides insight into how relational dynamics recover, or fail to recover, following systemic disruption.

As such, we sought to examine trust of community members in their CHWs in the aftermath of COVID-19 in three LMICs with different demographics, political stabilities, and CHW programme structures: Haiti, Malawi, and Rwanda. In particular, we considered how modifiable factors may impact community members’ trust levels in CHWs according to the framework. We were interested in two potentially modifiable factors. First, we wanted to explore the extent to which the sex of the CHW impacts the trust of the community members, with a particular focus on whether matching the sex of the CHW to the sex community member leads to greater trust. Second, we wanted to explore whether spending more time engaging with communities was associated with greater trust in CHWs. By investigating engagement between CHWs and their communities and their trust-related dynamics, we aim to gain valuable insights into opportunities to improve the effectiveness of CHW programmes.

## METHODS

### Study setting

We conducted a cross-sectional study in rural areas of Rwanda, Malawi, and Haiti that are supported by the non-profit organisation Partners In Health (PIH), known locally as *Zanmi Lasante* in Haiti, *Abwenzi Pa Za Umoyo* in Malawi, and *Inshuti Mu Buzima* in Rwanda. In all three countries, PIH provides direct support to local CHW programmes. In Haiti, PIH has supported the CHW programme in Lower Artibonite and the Central Plateau region since 1987. As of 2024, PIH supported approximately 2500 CHWs in Haiti, covering approximately 3.3 million people [21]. In alignment with Haiti’s national CHW programme, CHWs provide primary care, maternal and child healthcare, HIV and tuberculosis services, and more advanced care [21]. Notably, at the time of data collection, Haiti was experiencing substantial political turmoil, including pervasive street violence and frequent roadblocks, which made the work of CHWs exceedingly hazardous. In Malawi, PIH has been active in Neno District since 2005. As of 2023, PIH supported a network of 1233 CHWs in Malawi covering a population of approximately 234 600 people. In Malawi, PIH’s CHW programme uses a household model where a single CHW tends to all members of a household [22]. In Rwanda, PIH has worked in Burera, Kayonza, and Kirehe districts since 2005. As of 2024, PIH supported approximately 6000 CHWs in Rwanda, covering a population of approximately 1 108 878 people [9]. Rwanda’s national healthcare system incorporated CHWs in 2007, with each village having three CHWs, one focused on pregnancy, maternal and infant health, and a male-female pair who provide more general health services [23].

### Study design

We used baseline data collected as part of a larger cluster-randomised controlled trial assessing the effectiveness of targeted SMS messages to combat health misinformation among CHWs and their communities.

Each CHW was randomised to receive information about either COVID-19 or mental health in a particular messaging style over a duration of 12 months. For the trial, CHWs were recruited from 54 PIH-supported health facilities in Rwanda (n = 25), Malawi (n = 14), and Haiti (n = 15) and were eligible to participate if they served in the healthcare facility and were ≥18 years old. For the CHW longitudinal study, CHWs were randomly sampled from the facilities’ catchment areas, and the first 175 CHWs from each country who consented were enrolled (n = 525). The trial included two rounds of repeated cross-sectional surveys among community members living in the catchment areas served by the 525 sampled CHWs. This study is restricted to the baseline community member surveys.

### Recruitment

Community members were sampled in two stages. First, in each of the three countries, we randomly sampled 32 out of the 175 participating CHWs. Second, we identified community members residing in each of the communities supported by these CHWs. Our target sample size was 640 community members per country (n = 1920) (Appendix S2 in the **Online Supplementary Document**). The sample size was determined by a simulation study to ensure at ≥80% power to detect a 15% increase in vaccine intention within each country and >90% power to detect a 10% increase in intention to vaccinate. For logistical feasibility, each country chose the sampling strategy to recruit community members individually. In Haiti and Rwanda, eligible community members were recruited at neutral public locations unrelated to healthcare (*e.g.* a church hall, community hall, marketplace, or transport centre), with one neutral location identified for each of the 32 sampled communities. Potentially eligible individuals were informed of the study and invited to participate. Enrolment at each location occurred for 4–5 hours until the target sample size for each location was achieved. In Malawi, the 32 CHWs compiled a complete list of all the households in their catchment area, of which 20 households were randomly chosen. A community member within each selected household, ideally the household head, was recruited to participate in the study. While the recruitment at neutral public locations in Haiti and Rwanda reduced direct CHW influence on participation, it was logistically efficient and provided a broad exposure to community life. However, it might be biased toward mobile and socially active individuals and systematically exclude community subgroups who were engaged in wage labour or caregiving during the limited enrolment period. The recruitment strategy in Malawi might have included less visible populations, and estimates might have suffered from social desirability bias due to higher CHW involvement. These differences should be considered when interpreting cross-country comparisons of engagement and trust.

### Data collection

We surveyed respondents from June 2023 to October 2023. The survey was conducted in the local language for each country – Kinyarwanda in Rwanda, Chichewa in Malawi, and Haitian Creole in Haiti. Trained enumerators verbally administered the survey and directly entered data on tables into REDCap. The community member survey lasted 30–90 minutes and measured key demographic variables (*i.e.* age and sex), CHW engagement, and trust in the CHW.

All CHWs and community members provided informed consent before participating in the study. They received a baseline compensation between USD 5–10. The exact amount was country-specific and set by the country teams to align with national ethics committee guidelines. Furthermore, CHW received country- and time-specific compensation and reimbursement for travel when applicable. We de-identified and securely stored the data.

### Variable definitions

We measured the frequency of self-engagement with CHWs as the number of times in the past six months the community members had interacted with a CHW using a four-level Likert-type scale (1 = never, 2 = one time, 3 = 2–3 times, 4 = ≥4 times). Among community members who had interacted with a CHW at least once, we also measured how long a typical interaction lasted in minutes. Similarly, we measured the frequency of community engagement with CHWs by asking a community member how often in the past six months the community members had seen their CHW interact with the community using the same four-level Likert-type scale. Among community members who had observed at least one interaction, the community member was asked about the typical duration of these interactions in minutes. For our analysis, we estimated the cumulative six-month self-engagement and cumulative six-month community-engagement by multiplying visit frequency by visit duration to estimate the total time spent engaging with CHWs in the past six months. During these calculations, the frequency of self- and community-engagement was set conservatively by assuming the lower bound of the reported Likert level (1 = never set to 0, 2 = one time set to 1, 3 = 2–3 times set to 2, 4 = ≥4 times set to 4). We transformed the cumulative six-month self- and community-engagement into hours for reporting and analysis.

Among community members who reported having engaged with their CHW at least once, we measured trust between the community members and their CHW using the ten-item Trust-in-CHW Scale, which was originally validated in Bangladesh, Haiti, and Kenya [14]. The Trust-in-CHW Scale, developed by Sripad *et al.* [14], was selected because of its prior validation in LMIC settings, including Haiti. The Trust-in-CHW-Scale contains two sub-scales: healthcare competence and respectful communication. Answers to each item are given on a four-level Likert-type scale (1 = Never, 2 = Some of the time, 3 = Most of the time, 4 = All of the time). These two factors are key dimensions of trust, foundational to trust in healthcare systems [14,24]. To ensure contextual appropriateness, each country team translated and reviewed the scale to preserve its intended meaning and purpose. We also pilot-tested the surveys in each country before implementation. We estimated an overall trust score, a sub-score for healthcare competence, and a sub-score for respectful communication by averaging over the corresponding non-missing Likert-type scale responses to the Trust-in-CHW Scale. Missing answers for the items were averaged and imputed; as such only community members who did not answer any items have a missing score. The score and sub-scores were considered as continuous variables ranging from one to four, with higher values indicating greater trust.

### Data analysis

For analyses, we assumed missingness in covariates, engagement and trust score outcome was completely at random and conducted a complete case analysis. Due to the recruitment design of community members, groups of community members were cared for by the same CHW, introducing clustering. We reported descriptive statistics as percentages for categorical variables and used a Rao-Scott χ^2^ test to conduct global and pairwise comparisons by country while adjusting for CHW clustering. For continuous variables, we reported descriptive statistics as mean (x̄) and standard error (SE) and performed global and country-wise comparisons based on linear regression models across all three countries and country-specific linear regression models. We fit the linear regression models based on the ordinary-least-squares estimating equations and estimated survey-robust SEs. We formally tested for significant effects with clustering-adjusted Wald tests using a robust sandwich-type estimator for the covariance matrix. We explored associations between sex and cumulative self-engagement using a clustering-adjusted linear regression model that included three covariates: sex of the CHW, sex of the community members, and sex concordance between the CHW and the community member. Including all three terms in the same model allowed us to focus on whether matching the sex of the CHW to the sex of the community member, which is a modifiable factor, matters after adjusting for the sex of the CHW and sex of community member, which are not modifiable. We explored associations between sex and trust score using a similar clustering-adjusted linear regression model with covariates sex of the CHW, sex of the community member and sex concordance. We explored whether the duration of engagement with CHWs was associated with greater trust in CHWs by regressing trust score on cumulative self-engagement duration and cumulative community-engagement in two separate clustering-adjusted linear regression models. We established the significance of effects based on adjusted Wald tests using a robust sandwich-type estimator for the covariance matrix.

We used STATA, version 15.1 (StataCorp, College Station, Texas, USA) for all analyses and generated figures using *R*, version 4.3.2 (R Core Team, Vienna, Austria).

## RESULTS

### Community members

We interviewed 1920 community members, of whom we included 1901 who had complete data for sex, sex of their CHW, and frequency of self- and community-engagement with the CHW (Appendix S2 in the **Online Supplementary Document**). Overall, more than half (60.2%) of the community members were female (**Table 1**). However, most community members had a male CHW (60.5%), meaning that most community members were attended by a CHW of the opposite sex (54.8%). The highest discordance between the sex of the community members and the sex of CHW was in Malawi (60.2%). Community members were similar in age in Haiti (x̄ = 38.8), Malawi (x̄ = 37.4), and Rwanda (x̄ = 37.0).

**Table 1:** Description of the study population*

	Total (n = 1901)	Rwanda (n = 635)	Malawi (n = 636)	Haiti (n = 630)
**Sex**				
Male	756 (39.8)	314 (49.4)	160 (25.2)	282 (44.8)
Female	1145 (60.2)	321 (50.6)	476 (74.8)	348 (55.2)
**Age in years, x̄ (SE)†**	37.7 (0.44)	37.0 (0.74)	37.4 (0.71)	38.8 (0.85)
**Sex of assigned CHW**				
Male	751 (39.5)	217 (34.2)	199 (31.3)	335 (53.2)
Female	1150 (60.5)	418 (65.8)	437 (68.7)	295 (46.8)
**Concordance between the sex of the CHW and the community member**				
Different sexes	859 (45.2)	313 (49.3)	253 (39.8)	293 (46.5)
Matched sexes	1042 (54.8)	322 (50.7)	383 (60.2)	337 (53.5)

SE – standard error, x̄ – mean

*Values are presented as numbers (percentages) unless specified otherwise.

†Total number of available age data was 1865.

### Self- and community-engagement in the last six months

Approximately one-third (34.7%) of community members reported having never personally met their CHW in the past six months (**Table 2**). The proportion of respondents with no self-engagement with a CHW in the past six months was high in Haiti (51.1%) and Rwanda (47.2%), but only 5.8% in Malawi, where the majority (70.1%) of community members engaged ‘≥4 times’ with their CHW. Malawi reported significantly greater self-engagement than both Rwanda (*P* < 0.001) and Haiti (*P* < 0.001), while the difference was not significant between Rwanda and Haiti (*P* = 0.54). For community engagement, most community members in Malawi (76.1%) and Rwanda (57.3%) reported having observed an interaction between a community member and CHW ‘≥4 times’ in the past six months. In Haiti, 42.9% of community members observed no community engagement. The frequency of community engagement varied significantly by country, with Malawi reporting significantly greater community engagement than both Rwanda (*P* < 0.001) and Haiti (*P* < 0.001), and Rwanda reporting greater community engagement than Haiti (*P* < 0.001).

**Table 2:** Self- and community-engagement frequency among communities

	Total (n = 1901)	Rwanda (n = 635)	Malawi (n = 636)	Haiti (n = 630)	*P*-value*
**Self-engagement (past six months)**					<0.001†
Never	659 (34.7)	300 (47.2)	37 (5.8)	322 (51.1)	
One time	217 (11.4)	112 (17.6)	23 (3.6)	82 (13.0)	
2–3 times	361 (19.0)	122 (19.2)	130 (20.4)	109 (17.3)	
≥4 times	664 (34.9)	101 (15.9)	446 (70.1)	117 (18.6)	
**Community-engagement (past six months)**					<0.001‡
Never	414 (21.8)	99 (15.6)	45 (7.1)	270 (42.9)	
One time	118 (6.2)	28 (4.4)	17 (2.7)	73 (11.6)	
2–3 times	359 (18.9)	144 (22.7)	90 (14.2)	125 (19.8)	
≥4 times	1010 (53.1)	364 (57.3)	484 (76.1)	162 (25.7)	

CHW – community health worker

*χ^2^ test for pairwise and overall comparison, adjusted for clustering based on CHW sampling.

†The difference was significant between Rwanda and Malawi, and Malawi and Haiti, but not between Rwanda and Haiti (*P* = 0.54)

‡The difference was significant between all country combinations.

### Intensity of cumulative six-month self- and community-engagement duration

**Figure 1:**
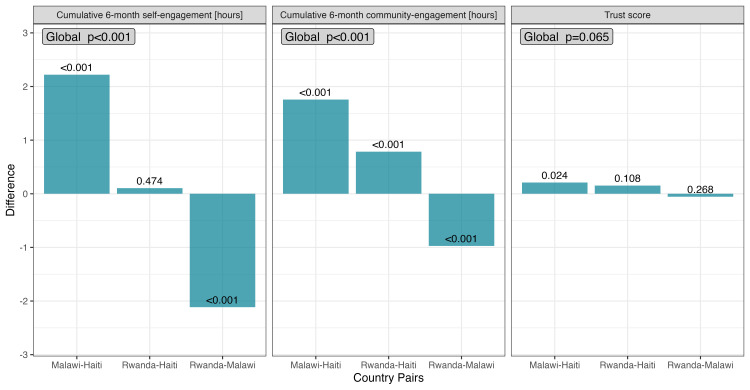
Mean difference in trust score and cumulative six-month self- and community-engagement between Rwanda, Malawi and Haiti.

We restricted our analyses of cumulative engagement and trust to community members who reported having interacted with their CHW at least once in the past six months and who did not have missing data on the Trust-in-CHW-Scale (n *=* 1212) (Appendix S2 in the **Online Supplementary Document**). The average cumulative six-month self-engagement duration (in hours) was highest in Malawi (x̄ *=* 3.3), followed by Rwanda (x̄ *=* 1.2) and Haiti (x̄ *=* 1.1) (**Table 3**). The self-engagement duration was significantly higher in Malawi compared to Haiti and Rwanda (*P* < 0.001), with no significant differences between Rwanda and Haiti (*P* = 0.47) (**Figure 1**). Similarly, the highest cumulative six-month community engagement duration (in hours) was observed among community members in Malawi (x̄ = 3.2), followed by those in Rwanda (x̄ = 2.2) and Haiti (x̄ = 1.4). Community members in Malawi reported having observed a significantly higher engagement duration between CHWs and their communities than between those in Rwanda and Haiti (*P* < 0.001). The community-engagement duration in Rwanda was significantly larger than in Haiti (*P* < 0.001).

**Table 3:** Average duration of cumulative six-month self- and community-engagement and average trust score*

	Total (n = 1212)	Rwanda (n = 332)	Malawi (n = 587)	Haiti (n = 293)
**Cumulative six-month self-engagement duration (in hours)**	2.2 (0.08)	1.2 (0.10)	3.3 (0.14)	1.1 (0.11)
**Cumulative six-month community-engagement duration (in hours)**	2.5 (0.08)	2.2 (0.13)	3.2 (0.12)	1.4 (0.20)
**Trust score**	3.5 (0.03)	3.5 (0.04)	3.6 (0.03)	3.3 (0.09)
Healthcare competence	3.5 (0.03)	3.5 (0.04)	3.5 (0.04)	3.3 (0.09)
Respectful communication	3.6 (0.03)	3.5 (0.04)	3.7 (0.03)	3.4 (0.08)

*Values are presented as means (standard errors).

### Trust score

Among community members who had interacted at least once with a CHW in the last six months, overall, the trust of community members in their CHW was high across all three countries. The average trust score was the highest in Malawi (x̄ = 3.6), followed by Rwanda (x̄ = 3.5) and Haiti (x̄ = 3.3). The trust of community members in their CHWs was significantly higher in Malawi than in Haiti (*P* = 0.02), while the trust score did not vary significantly between Rwanda and Malawi (*P* = 0.27), and Rwanda and Haiti (*P* = 0.11) (**Figure 1**). The distribution of the trust score exhibits right-skewness and a ceiling effect, particularly in Malawi and Rwanda (Appendix S3 in the **Online Supplementary Document**).

### Association between sex and engagement, and sex and trust

We considered the association between the cumulative six-month self-engagement and being female, being treated by a female CHW, and being sex concordant with one’s CHW. Across covariates, we could not detect any clear association between the sex variables and engagement, as well as the sex variables and trust. The associations between sex and self-engagement duration varied substantially by country (Appendix S4 and S5 in the **Online Supplementary Document**). Sex concordance had a non-significant positive association with self-engagement duration in Malawi and Haiti, but a negative effect in Rwanda. Similarly, when we investigated the associations between being female, being treated by a female CHW, and having the same sex as one’s CHW with trust, we observed small, non-significant associations. Sex concordance had a non-significant positive association with the trust score in Malawi and Rwanda, but a negative effect in Haiti. Additional analyses of sex effects on engagement frequency and trust, while providing a more nuanced insight, did not detect any clear association. Additionally, the average trust score and self- and community-engagement duration seemed consistent across age groups. The sensitivity analysis based on the corresponding ordinal trust score outcome corroborates these results (Appendices S8–11 in the **Online Supplementary Document**).

### Association between trust score and cumulative six-month self- and community engagement duration

Cumulative six-month self-engagement between a community member and their CHW and the cumulative six-month community-engagement were significantly associated with increased trust score for all countries among community members who interacted with a CHW at least once in the past six months (Appendices S6 and S7 in the **Online Supplementary Document**). The magnitude of the association between self-engagement and trust was mostly higher than the magnitude of the association between community-engagement and trust. Both self-engagement duration (regression coefficient b = 0.19) and community-engagement duration (b = 0.11) associations were most pronounced in Haiti, which had the lowest levels of engagement. Conversely, both self-engagement duration (b = 0.03) and community-engagement duration (b = 0.03) associations were the least pronounced in Malawi, where engagement was the highest.

These results were corroborated by the sensitivity analysis, which was based on an ordinal regression of the trust score and the self-engagement duration (Appendix S11 in the **Online Supplementary Document**).

## DISCUSSION

We investigated community members’ engagement and trust in CHWs after the COVID-19 pandemic in rural villages in Haiti, Rwanda, and Malawi, based on baseline survey data from a larger cluster-randomised controlled trial. The results reported in this paper provide insight into the existing relationship between CHWs and community members in terms of engagement frequency, duration, and trust and serve as a benchmark for post-intervention comparisons in the larger trial.

We conducted the study in communities supported by PIH, where interaction between CHWs and their communities has historically been reported as high.

However, even in this context, our data showed that most community members in Haiti and Rwanda reported no interaction with their CHWs in the past six months. Descriptive statistics (not included in this paper) suggested that non-interaction is observed across age and sex strata, and CHWs do not seem to target patient subpopulations (*e.g.* women of reproductive age) systematically. This finding may suggest that community members observed in this study interact less with their CHW than is commonly the case. Such a general engagement gap between CHWs and their communities may hinder the potential impact of CHW programmes [16], including for disease prevention efforts [19,25]. Interestingly, this finding was consistent in Haiti, which was experiencing extreme political instability during the time of data collection, and in Rwanda, which had no political or security issues at the time of data collection. This observation warrants a more structured investigation of non-interaction rates and potential reasons among communities and CHWs.

Across the diverse settings of Rwanda, Haiti, and Malawi, community members who had interacted with their CHW in the past six months reported high trust in their CHW. Both longer duration of self-engagement and longer perceived duration of community-engagement were significantly associated with increased trust in all three countries. This finding aligns with previous reports that trust increases as the frequency of engagement between a community member and their CHW increases [10,17]. CHWs become trusted reference persons who provide health-related information and advice [13,26] and function as a necessary support system [10]. Importantly, the magnitude of the association between engagement and trust was greater when we looked at the duration of an individual’s personal interactions with the CHW than when we considered the perceived duration of community engagement. The magnitude of association was also the strongest in the countries that reported the lowest durations of engagement. These findings underscore the importance of designing CHW programmes to allow for meaningful interactions between CHWs and individual community members.

Previous literature suggested that both the sex of CHWs and matching the sex of CHWs to their community members might influence trust, communication, and health-seeking behaviour [10,12,15]. We did not find that these factors were associated with either engagement or trust score. This finding aligns with results from the validation study for the Trust-in-CHW scale, which also did not show a sex effect [14]. Notably, previous papers that did observe an association between sex and trust assessed different dimensions of trust, including trust in a CHW’s confidentiality and the acceptance of a vulnerable situation relying on the trustor’s care for the trustee [12,15]. The sex of the CHW or sex matching between CHWs and community members may matter more for these dimensions of trust than for trust in healthcare competence and respectful communication, which are the two areas of focus for the Trust-in-CHW scale.

The comparative analysis highlights important differences among the three countries. Malawi demonstrated higher levels of CHW engagement and trust than Haiti and Rwanda. This could reflect methodological differences in sampling. Unlike Haiti and Rwanda, where community members were recruited at high-traffic public venues, Malawi supplemented their sample with additional community members who were sampled directly from a list of households developed by the CHWs. This list of households did not necessarily represent the general community and may have been biased towards households with whom the CHW had more frequent engagement or more positive relationships. This bias limits the comparability of engagement statistics across countries and implies that we likely overstated the Malawi CHW programme’s community-wide engagement levels. However, the higher engagement in Malawi may also be attributable to the structure of the Malawian CHW programme. In 2007, PIH implemented the Household Model in Neno District [22]. In this model, CHWs are assigned to oversee all members of a household – irrespective of their health status – to promote good nutrition and growth monitoring, sanitation and hygiene, general vaccination and disease monitoring, and link families to essential services [27,28]. These close and consistent interactions with larger households likely foster deeper ties and strong trust between families and their CHW and may also decrease stigmatisation and discrimination and facilitate comprehensive and sustainable community-wide patient healthcare [16,22].

Conversely, estimates of trust and engagement in Haiti and Rwanda may be influenced by the recruitment at health-neutral public locations. This approach may have preferentially included community members who were able to leave their homes and who were not engaged in wage labour or caregiving during the recruitment period.

### Limitations

This study has several limitations. First, we conducted the study in rural areas in Malawi, Rwanda, and Haiti, in which PIH’s continued engagement in maintaining access to healthcare has fostered relatively deep trust within village communities over decades. This limits the generalisability of our findings to other rural communities. Additionally, in the PIH context specifically, factors surrounding CHW training, supply availability, health system experience, and community health literacy are standardised and might differ from CHW care in other settings, where training and supply are more localised. However, the CHWs belonged to different PIH-supported facilities serving wider geographic areas within each country. Positive associations between trust and self- and community-engagement were consistently established across countries and areas. Second, the household-based recruitment strategy in Malawi likely led to a Malawian sample that is more engaged with and more trusting of their CHWs than the general population. Third, we measured trust using the ten-item Trust-in-CHW Scale, which focuses on only two dimensions (*i.e.* competence and communication) of the complex phenomenon of trust. Previous studies have considered additional dimensions of trust, such as trust in a CHW’s confidentiality regarding sensitive personal health information [29] and trust that the trustee will care for the trustor’s interests [15]. Additionally, the ten-item Trust-in-CHW Scale was validated before the COVID-19 pandemic, while we applied it in a post-pandemic context. Furthermore, we relied on self-reported measures, enumerators associated with PIH assessments, and financial compensation. All these factors might introduce recall or social desirability bias to our data. Also, trained neutral enumerators associated with PIH questioned the community members, yet they were previously unknown to the community. The community members were interviewed in neutral locations in Rwanda and Haiti, while in Malawi, they were interviewed in their homes. Consequently, across countries, the data collection process provided sufficient neutrality to consider the responses to be honest. Moreover, we did not assess the trust levels of community members who reported having never engaged with CHWs since we did not expect a large proportion of the population to be disengaged from their CHW. Therefore, our estimates of community trust in CHWs may be overestimated, especially if individuals who do not trust CHWs systematically choose not to engage with them. Finally, we used a cross-sectional design and therefore cannot draw any conclusions about the directionality of the relationships assessed. Also, we did not assess the CHWs' perspectives on their relationships with community members. Engagement and trust are not unidirectional – future research should consider reciprocal perspectives.

## CONCLUSIONS

Across three different countries with substantially different CHW programmes, we observed that the duration of engagement with a CHW was positively associated with a community member’s trust in CHWs. However, we also observed that over a third of the population had no meaningful engagement with their CHWs. This finding highlights the importance of structuring CHW programmes to empower CHWs to invest sufficient time into engaging with their communities. Task shifting, undefined role expectations, and inadequate compensation often leave CHWs stretched thin [5,6], undermining their ability to engage meaningfully with their communities. Previous research has identified that CHWs require sufficient training in key skills, medical supplies and equipment, competent supervision, and adequate compensation for their essential work in providing healthcare [30]. Additionally, monitoring frequency and duration of interactions between CHWs and community members may be an easily measurable indicator for assessing the quality of a CHW system. We also found that matching the sex of the CHW to the community member they serve may not provide improvement in trust. However, because trust is a complex and multidimensional construct, more research is required to fully understand sex-related dynamics and the importance of regular, high-quality CHW-community engagement in building trusting relationships between CHWs and community members.

## Additional material


Online Supplementary Document
