# Supplementary material for: Measuring community engagement and trust in community health workers in Haiti, Malawi, and Rwanda: a cross-sectional study
Source: J Glob Health. 2026 Jul 24;16:04217. doi: 10.7189/jogh.16.04217 (PMC13397096; doi:10.7189/jogh.16.04217)
Supplement: Online Supplementary Document [file jogh-16-04217-s001.pdf]

Supplement to: Armbruster S, Joseph S, Dubique K, Baganizi E, Gondwe F, Belizaire N, Chery M, Faure P, Musafiri T, Chabwera B, Mugunga J, Clisbee M, Barnhart D, Hedt-Gauthier B, Munyaneza F, Measuring community engagement and trust in community health workers in Haiti, Malawi, and Rwanda: a cross-sectional study, J Glob Health. 2026;16:04217.

Appendix – Sample size, sex-interactions for trust score, self- and community-interaction:

Appendix S1: Directed Acyclic Graph for Trust-Engagement Relationship between community members and CHWs.

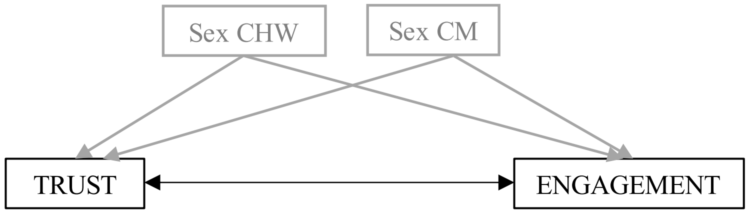

Appendix S2. Flowchart of sample sizes based on conditional questioning and missingness.

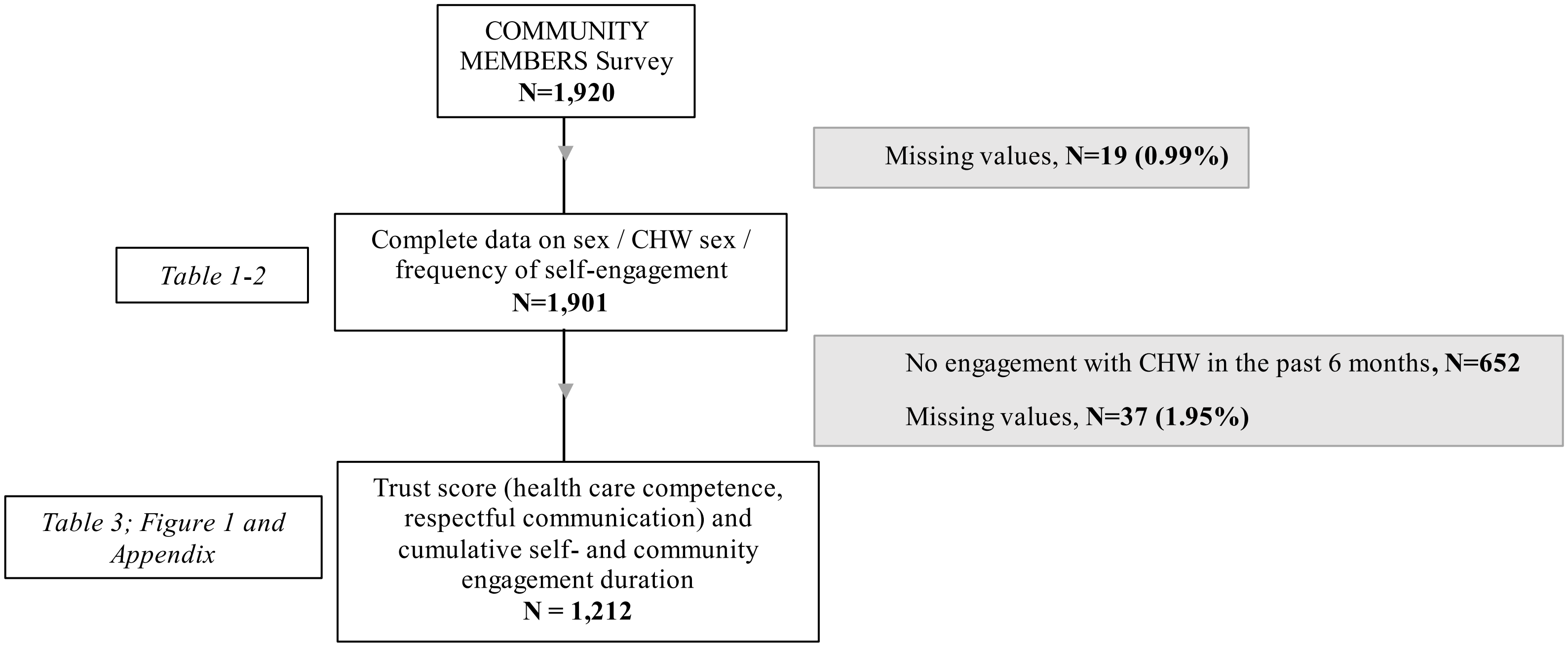

Appendix S3. Histogram of trust score for community members, stratified by country.

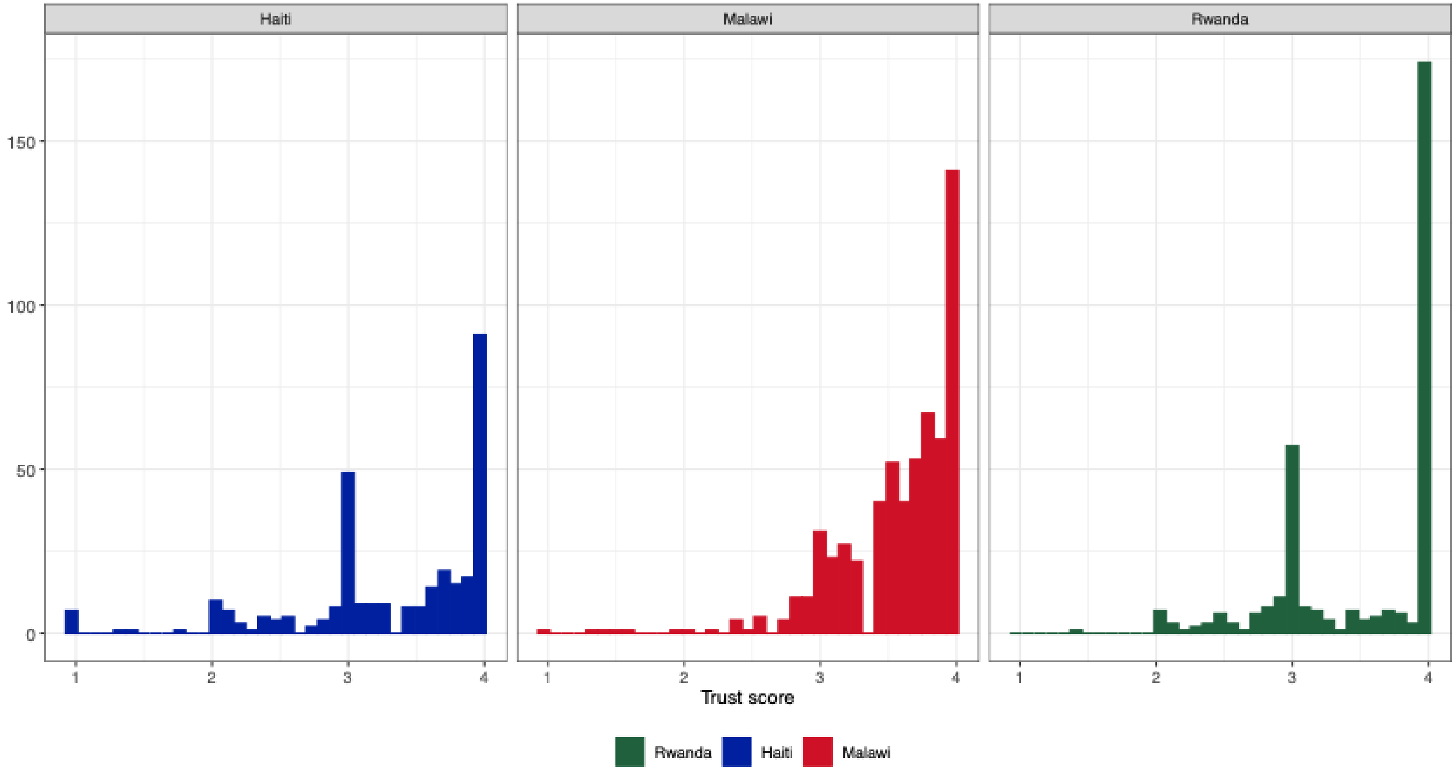

<u>Appendix S4:</u> (A) Country-specific expected effect of having a female CHW, being female and sex concordance on cumulative 6-month self-engagement duration [hours]; simultaneous estimation corrected for clustering by CHW. **(B)** Country-specific expected effect of having a female CHW, being female and sex concordance on trust score; simultaneous estimation corrected for clustering by CHW. (N=1,212)

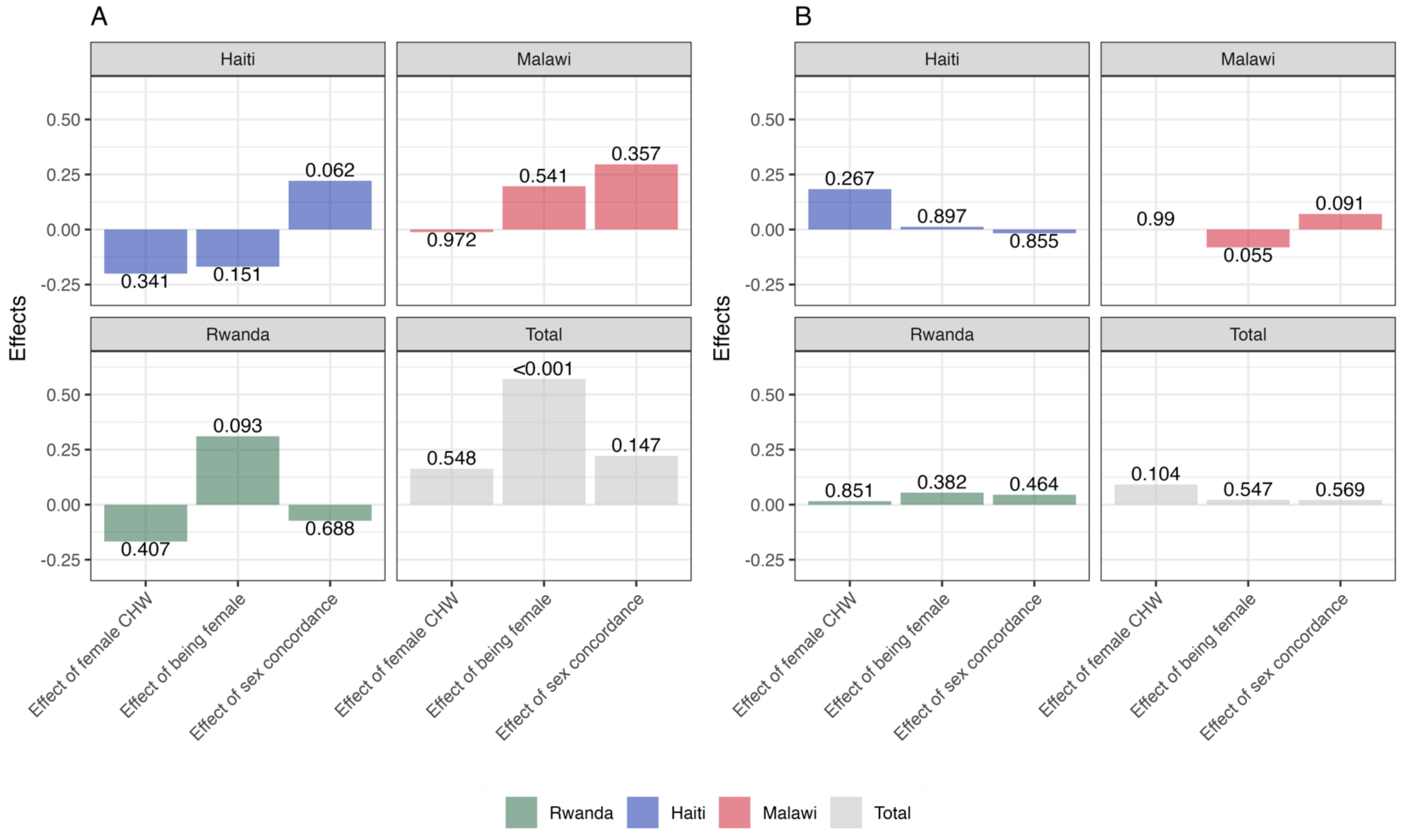

<u>Appendix S5:</u> Estimated regression coefficients with self-engagement duration and trust score as dependent variables

	Total (n = 1212)			Rwanda (n = 332)			Malawi (n = 587)			Haiti (n = 293)		
	**n**	***b* (95% CI)**	***P*-value***	**n**	***b* (95% CI)**	***P*-value***	**n**	***b* (95% CI)**	***P*-value***	**n**	***b* (95% CI)**	***P*-value***
**Self-engagement duration (in hours)**												
Female community member	779	0.57 (0.27, 0.87)	<0.001	178	0.31 (−0.05, 0.68)	0.093	439	0.2 (−0.45, 0.84)	0.541	162	−0.17 (−0.40, 0.06)	0.151
Female CHW	761	0.16 (−0.37, 0.7)	0.548	218	−0.17 (−0.57, 0.24)	0.407	405	−0.01 (−0.81, 0.69)	0.972	138	−0.20 (−0.62, 0.22)	0.341
Sex concordance	684	0.22 (−0.08, 0.52)	0.147	172	−0.07 (−0.44, 0.29)	0.688	355	0.3 (−0.35, 0.94)	0.357	157	0.22 (−0.01, 0.46)	0.062
**Trust score**												
Female community member	779	0.02 (−0.05, 0.09)	0.547	178	0.05 (−0.07, 0.18)	0.382	439	−0.08 (−0.16, 0.002)	0.055	162	0.01 (−0.18, 0.21)	0.897
Female CHW	761	0.09 (−0.02, 0.2)	0.104	218	0.02 (−0.15, 0.18)	0.851	405	0.0007 (−0.11, 0.11)	0.990	138	0.18 (−0.15, 0.52)	0.267
Sex concordance	684	0.02 (−0.05, 0.09)	0.569	172	0.05 (−0.08, 0.17)	0.464	355	0.07 (−0.01, 0.15)	0.091	157	−0.02 (−0.21, 0.18)	0.855

CHW – community health worker, CI – confidence interval

**P*-values are based on clustering-adjusted marginal Wald test.

Appendix S6:

Country-specific effect of cumulative 6-month self- and community-engagement [hours] on trust score; estimation corrected for clustering by CHW. (N=1,212)

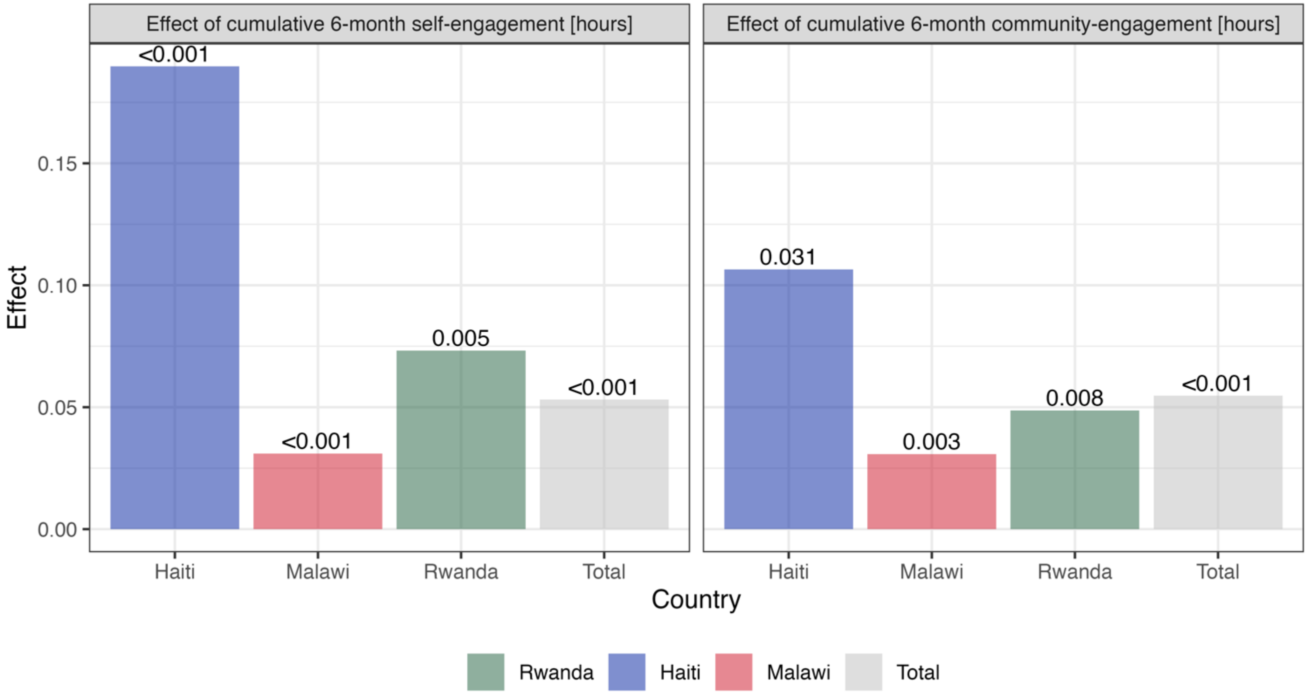

Appendix S7. Estimated regression coefficients with trust score as a dependent variable

	**Total (n = 1212)**		**Rwanda (n = 332)**		**Malawi (n = 587)**		**Haiti (n = 293)**	
	***b* (95% CI)**	***P*-value***	***b* (95% CI)**	***P*-value***	***b* (95% CI)**	***P*-value***	***b* (95% CI)**	***P*-value***
Self-engagement duration (in hours)	0.05 (0.04, 0.07)	<0.001	0.07 (0.02, 0.12)	0.005	0.03 (0.01, 0.05)	<0.001	0.19 (0.09, 0.29)	<0.001
Community-engagement duration (in hours)	0.05 (0.03, 0.08)	<0.001	0.05 (0.01, 0.08)	0.008	0.03 (0.01, 0.05)	0.003	0.11 (0.01, 0.2)	0.031

CI – confidence interval
**P*-values are based on clustering-adjusted marginal Wald test.

Appendix S8. Self- and community-engagement frequency (N=1,901), cumulative self- and community-engagement and trust score (N=1,212); stratification by sex for community members and CHW and country.

	Rwanda				Malawi				Haiti			
Sex of community members	**Male**		**Female**		**Male**		**Female**		**Male**		**Female**	
Sex of assigned CHW	*male*	*female*	*male*	*female*	*male*	*female*	*male*	*female*	*male*	*female*	*male*	*female*
	**N=109**	**N=205**	**N=108**	**N=213**	**N=53**	**N=107**	**N=146**	**N=330**	**N=162**	**N=120**	**N=173**	**N=175**
**Self-engagement frequency**												
Never	54 (49.5%)	104 (50.7%)	48 (44.4%)	94 (44.1%)	3 (5.7%)	7 (6.5%)	8 (5.5%)	19 (5.8%)	83 (51.2%)	62 (51.7%)	87 (50.3%)	90 (51.4%)
One time	24 (22.0%)	35 (17.1%)	18 (16.7%)	35 (16.4%)	2 (3.8%)	4 (3.7%)	6 (4.1%)	11 (3.3%)	17 (10.5%)	19 (15.8%)	16 (9.2%)	30 (17.1%)
Two or three times	18 (16.5%)	37 (18.0%)	22 (20.4%)	45 (21.1%)	7 (13.2%)	25 (23.4%)	36 (24.7%)	62 (18.8%)	27 (16.7%)	22 (18.3%)	40 (23.1%)	20 (11.4%)
Four or more times	13 (11.9%)	29 (14.1%)	20 (18.5%)	39 (18.3%)	41 (77.4%)	71 (66.4%)	96 (65.8%)	238 (72.1%)	35 (21.6%)	17 (14.2%)	30 (17.3%)	35 (20.0%)
**Community-engagement frequency**												
Never	16 (14.7%)	27 (13.2%)	17 (15.7%)	39 (18.3%)	1 (1.9%)	4 (3.7%)	18 (12.3%)	22 (6.7%)	61 (37.7%)	55 (45.8%)	77 (44.5%)	77 (44.0%)
One time	4 (3.7%)	10 (4.9%)	4 (3.7%)	10 (4.7%)	0 (0.0%)	1 (0.9%)	6 (4.1%)	10 (3.0%)	14 (8.6%)	14 (11.7%)	16 (9.2%)	29 (16.6%)
Two or three times	22 (20.2%)	48 (23.4%)	26 (24.1%)	48 (22.5%)	4 (7.5%)	14 (13.1%)	23 (15.8%)	49 (14.8%)	35 (21.6%)	23 (19.2%)	41 (23.7%)	26 (14.9%)
Four or more times	67 (61.5%)	120 (58.5%)	61 (56.5%)	116 (54.5%)	48 (90.6%)	88 (82.2%)	99 (67.8%)	249 (75.5%)	52 (32.1%)	28 (23.3%)	39 (22.5%)	43 (24.6%)
	**N=54**	**N=100**	**N=60**	**N=118**	**N=49**	**N=99**	**N=133**	**N=306**	**N=75**	**N=56**	**N=80**	**N=82**
**Cumulative 6-month self-engagement duration; mean (s.e.)**	1.1 (0.11)	1.0 (0.15)	1.5 (0.27)	1.2 (0.19)	3.3 (0.53)	3.0 (0.26)	3.2 (0.22)	3.4 (0.20)	1.4 (0.20)	0.9 (0.18)	1.0 (0.15)	1.0 (0.15)
**Cumulative 6-month community-engagement duration; mean (s.e.)**	2.2 (0.24)	2.2 (0.21)	2.0 (0.12)	2.3 (0.25)	3.5 (0.63)	3.2 (0.22)	2.8 (0.25)	3.3 (0.14)	1.9 (0.32)	1.0 (0.20)	1.2 (0.18)	1.4 (0.48)
**Trust score; mean (s.e.)**	3.5 (0.08)	3.5 (0.07)	3.5 (0.08)	3.6 (0.05)	3.7 (0.05)	3.6 (0.05)	3.5 (0.04)	3.6 (0.05)	3.3 (0.07)	3.5 (0.13)	3.3 (0.16)	3.5 (0.15)
Health care competence	3.5 (0.09)	3.5 (0.08)	3.5 (0.09)	3.5 (0.05)	3.6 (0.06)	3.5 (0.06)	3.4 (0.06)	3.5 (0.05)	3.2 (0.08)	3.5 (0.13)	3.2 (0.17)	3.5 (0.16)
Respectful communication	3.5 (0.08)	3.5 (0.07)	3.5 (0.09)	3.6 (0.05)	3.7 (0.05)	3.7 (0.05)	3.7 (0.04)	3.7 (0.04)	3.3 (0.08)	3.5 (0.13)	3.4 (0.16)	3.5 (0.14)

Appendix S9. Statements on community member trust in their CHW by statement, stratified by sex of CHW and community member. (N=1,212)

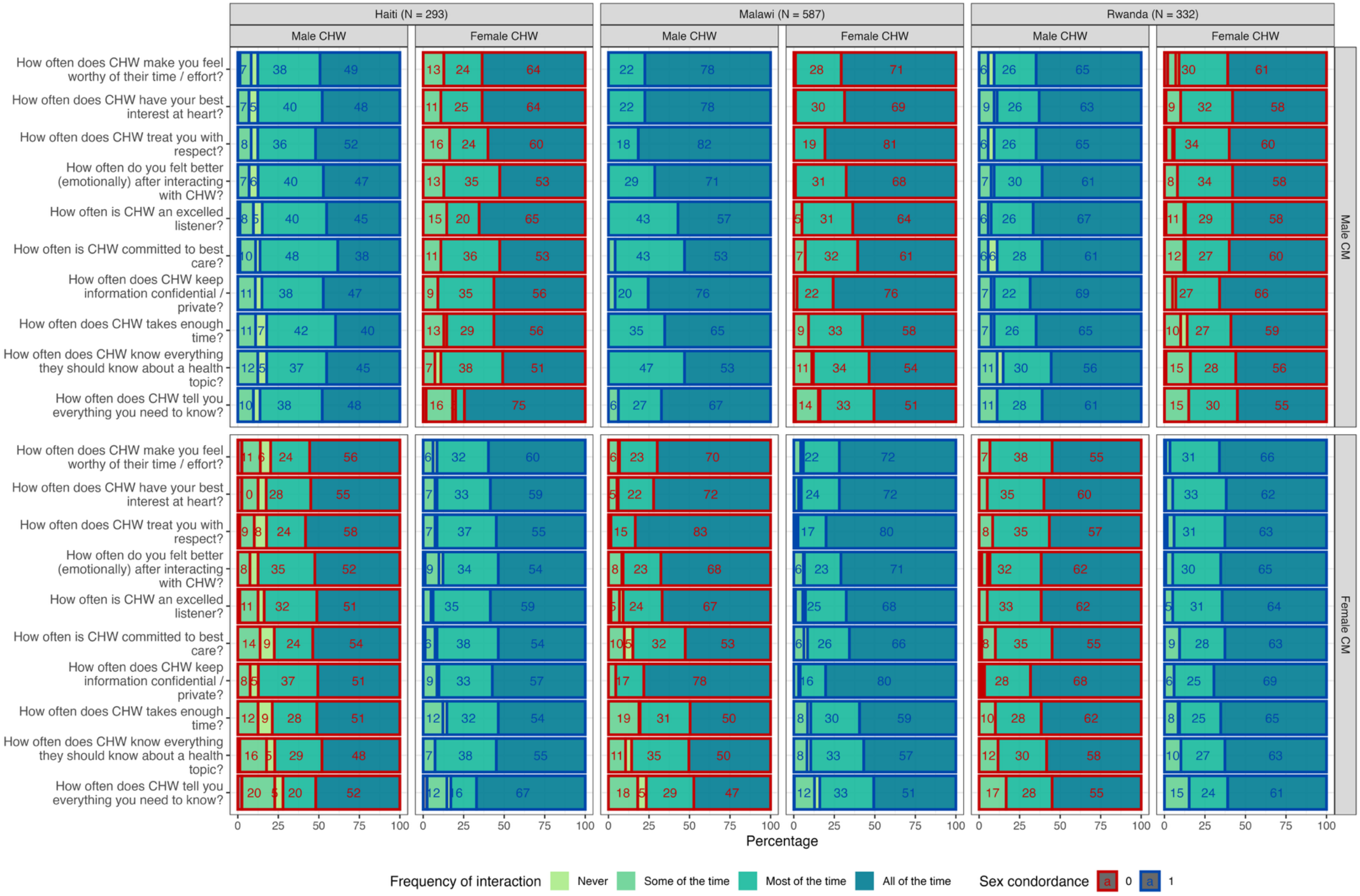

Appendix S10. Mean trust score and self-engagement duration [hours] (N = 1,192; 20 community members are missing age information); stratified by country and age group [years]

	**Total**					
Age group [years]	*All ages*	*19-29*	*30-55*	*56-65*	*66-85*	*>85*
	**N=1,192**	**N=392**	**N=648**	**N=102**	**N=47**	**N=3**
**Trust score**	3.5 (0.02)	3.5 (0.03)	3.5 (0.02)	3.5 (0.05)	3.5 (0.09)	3.6 (0.35)
**Cumulative 6-month self-engagement duration; mean (s.e.)**	2.2 (0.06)	2.4 (0.12)	2.1 (0.08)	1.9 (0.18)	2.1 (0.26)	1.2 (0.46)
**Cumulative 6-month community-engagement duration; mean (s.e.)**	2.5 (0.06)	2.5 (0.12)	2.6 (0.08)	2.0 (0.16)	2.1 (0.22)	0.8 (0.4)
	**Rwanda**					
	All ages	*19-29*	*30-55*	*56-65*	*66-85*	*>85*
	**N=332**	**N=98**	**N=204**	**N=25**	**N=4**	**N=1**
**Trust score**	3.5 (0.03)	3.5 (0.06)	3.5 (0.04)	3.5 (0.12)	3.5 (0.25)	3.0 (.)
**Cumulative 6-month self-engagement duration; mean (s.e.)**	1.2 (0.08)	1.3 (0.19)	1.1 (0.08)	0.9 (0.14)	2.9 (1.75)	0.5 (.)
**Cumulative 6-month community-engagement duration; mean (s.e.)**	2.2 (0.1)	2.2 (0.24)	2.2 (0.12)	1.6 (0.22)	2.5 (0.3)	1.3 (.)
	**Malawi**					
	All ages	*19-29*	*30-55*	*56-65*	*66-85*	*>85*
	**N=569**	**N=205**	**N=292**	**N=43**	**N=28**	**N=1**
**Trust score**	3.6 (0.02)	3.6 (0.03)	3.6 (0.03)	3.6 (0.08)	3.5 (0.09)	3.9 (.)
**Cumulative 6-month self-engagement duration; mean (s.e.)**	3.3 (0.1)	3.5 (0.17)	3.3 (0.13)	3.1 (0.34)	2.4 (0.34)	2.0 (.)
**Cumulative 6-month community-engagement duration; mean (s.e.)**	3.2 (0.09)	3.2 (0.15)	3.3 (0.13)	2.8 (0.29)	2.6 (0.30)	0.0 (.)
	**Haiti**					
	All ages	*19-29*	*30-55*	*56-65*	*66-85*	*>85*
	**N=291**	**N=89**	**N=152**	**N=34**	**N=15**	**N=1**
**Trust score**	3.4 (0.04)	3.3 (0.07)	3.4 (0.06)	3.5 (0.07)	3.4 (0.23)	4.0 (.)
**Cumulative 6-month self-engagement duration; mean (s.e.)**	1.1 (0.06)	1.0 (0.11)	1.1 (0.09)	1.1 (0.14)	1.3 (0.23)	1.0 (.)
**Cumulative 6-month community-engagement duration; mean (s.e.)**	1.4 (0.09)	1.2 (0.14)	1.6 (0.15)	1.3 (0.19)	0.9 (0.21)	1.2 (.)

<u>Appendix S11.</u> Count for ordinal trust score across countries and results for ordinal regression for ordinal trust score, reported in terms of estimates for the log odds ratio and 95% confidence interval.

	**Rwanda**	**Malawi**	**Haiti**
	**N = 332**	**N = 587**	**N = 293**
	N (%)	N (%)	N (%)
**Ordinal trust score**			
1) ≤3.1	114 (34%)	95 (16%)	108 (37%)
2) 3.1 < … < 4	44 (13%)	351 (60%)	96 (33%)
3) ≥ 4	174 (52%)	141 (24%)	89 (30%)
**Ordinal regression: trust - sex**	**Log odds ratio (95% CI)**	**Log odds ratio (95% CI)**	**Log odds ratio (95% CI)**
Effect of being female	0.13 (-0.29, 0.55)	-0.15 (-0.53, 0.22)	0.13 (-0.52, 0.78)
Effect of female CHW	0.09 (-0.349, 0.67)	0.03 (-0.48, 0.53)	0.4 (-0.54, 1.35)
Effect of sex concordance	0.1 (-0.33, 0.52)	0.25 (-0.13, 0.62)	-0.15 (-0.8, 0.49)
Ordinal regression: trust - engagement	**Log odds ratio (95% CI)**	**Log odds ratio (95% CI)**	**Log odds ratio (95% CI)**
Effect of self-engagement duration [hours]	0.21 (-0.11, 0.54)	0.1 (0.03, 0.17)	0.52 (0.2, 0.84)
